# Sri Lanka Society of Gastroenterology Collaborative Hepatocellular Carcinoma Snapshot Audit (SLSG CARSA): A Nationwide, Cross‐Sectional Snapshot Study

**DOI:** 10.1002/cnr2.70746

**Published:** 2026-11-05

**Authors:** Madunil Anuk Niriella, Viranga Sathsarani Ranathunga, Sanduni Nirasha, Rohan Chaminda Siriwardana, Kuleesha Senarath Kodisinghe, Sujeewa Ravindu Kumarasena, Tharanga Liyanaarchchi, Suchintha Bandara Tillakaratne, Hasitha Wijewantha, Uditha Bandara Dassanayake, Vithiya Rishikesawan, Chamila Kumara Ranawaka, Anuradha Kajendran, Karthiha Balendran, Sanjeewa Aryasingha, Duminda Subasinghe, Nilesh Fernandopulle, Sudual Mananjala Senanayake, Anuradha Supun Dassanayake, Ishan de Zoysa, Chinthaka Viduranga de Silva, Chamila Subasinghe, Sumedha Udumalagala, Supun Kulathunga, Tilan Aponso, Vadivel Vijitharan, Isuru Upanishad, Nandana Dinamithra, Arunasalam Pathmeswaran, Hithanadura Janaka de Silva

**Affiliations:** ^1^ Colombo North Centre for Liver Disease, Faculty of Medicine University of Kelaniya Ragama Sri Lanka; ^2^ Research Support Centre Sri Lanka Society of Gastroenterology Colombo Sri Lanka; ^3^ District General Hospital Matara Sri Lanka; ^4^ National Hospital Galle Sri Lanka; ^5^ National Hospital Kandy Sri Lanka; ^6^ District General Hospital Chilaw Sri Lanka; ^7^ Colombo North Teaching Hospital Ragama Sri Lanka; ^8^ National Hospital Jaffna Sri Lanka; ^9^ Department of Medicine, Faculty of Medicine University of Jaffna Jaffna Sri Lanka; ^10^ National Hospital Colombo Sri Lanka; ^11^ Department of Surgery, Faculty of Medicine University of Colombo Colombo Sri Lanka; ^12^ Colombo South Teaching Hospital Kalubovila Sri Lanka; ^13^ Teaching Hospital Anuradhapura Sri Lanka; ^14^ Teaching Hospital Ratnapura Sri Lanka; ^15^ Teaching Hospital Kalutara Sri Lanka; ^16^ Teaching Hospital Kuliyapitiya Sri Lanka; ^17^ Sri Lanka Army Hospital Colombo Sri Lanka; ^18^ Teaching Hospital Batticaloa Sri Lanka; ^19^ Teaching Hospital Pollonnaruwa Sri Lanka

**Keywords:** cross‐sectional survey, HCC, hepatocellular carcinoma, incidence, liver cancer, snapshot study, Sri Lanka

## Abstract

**Background and Aims:**

Sri Lanka lacks nationwide data on the incidence and characteristics of hepatocellular carcinoma (HCC); there is no prospective national HCC registry, and the only published clinical series comes from a single tertiary centre. We conducted a three‐month, nationwide, snapshot cross‐sectional survey to address this gap.

**Methods:**

We invited clinicians at all state‐sector and major private‐sector institutions providing gastroenterology, gastrointestinal surgery, or oncology services in the nine provinces of Sri Lanka to report new cases diagnosed between June and August 2024. Diagnosis required either dynamic contrast‐enhanced imaging in cirrhotic patients or histology in non‐cirrhotic cases, or when imaging was inconclusive. We collected data on demographics, performance status, liver disease, risk factors, tumour characteristics and treatment approaches using a REDCap electronic case‐report form with built‐in validation and query resolution. Age‐specific counts were multiplied by four to annualise the three‐month observation and standardised to the WHO World Standard Population. Confidence intervals (CI) were derived from the observed (unannualised) case count, and sensitivity analyses examined seasonality and incomplete ascertainment. Apart from the incidence estimates, the analysis was descriptive, and no hypothesis testing was undertaken. The study is reported in accordance with STROBE.

**Results:**

A total of 140 patients were included. The age‐standardised incidence rate was 2.51/100000 per year (95% CI 2.11–2.97; crude rate 2.54/100000, 95% CI 2.13–2.99). Most patients (78.6%) were male, with a mean age of 67.4 years. Key findings were as follows: the most frequently recorded risk factors were diabetes (66.4%) and unsafe alcohol use (62.9%); 85.7% had underlying cirrhosis, mostly Child‐Turcotte‐Pugh class A (62.5%); 43.8% presented with single nodules (*n* = 137), predominantly 2–5 cm (45.5%; *n* = 134); 16.4% showed tumour invasion and 12.9% had extra‐hepatic spread; most cases were BCLC stages A (27.9%) or B (30.7%). No positive HBsAg or anti‐HCV results were documented among those tested. Only 46.4% of cases were recorded as screen‐detected, and an initial treatment decision was recorded for only 55.0% of cases.

**Conclusion:**

This survey provides the first national estimate of HCC incidence in Sri Lanka, approximately twice the previous estimate. Because the earlier figure was derived by modelling in the absence of national surveillance, this difference should be interpreted as evidence of a previously underestimated burden, at least partly attributable to improved case ascertainment, rather than as a demonstrated temporal rise. HCC primarily affects older males with cirrhosis; most patients presented with preserved liver function and moderately sized single tumours at BCLC stages A/B. Strengthened national surveillance, structured early detection strategies and a prospective national HCC registry are needed.

**Abbreviations**
ASR: age‐standardised rate
AUDIT: alcohol use disorders identification test
BCLC: Barcelona Clinic Liver Cancer
BMI: Body Mass Index
CI: confidence interval
CTP: Child‐Turcotte‐Pugh
HBV: hepatitis B virus
HCC: hepatocellular carcinoma
HCV: hepatitis C virus
NAFLD: non‐alcoholic fatty liver disease
NIC: National Identity Card
PEI: percutaneous ethanol injection
REDCap: Research Electronic Data Capture
SLSG CARSA: Sri Lanka Society of Gastroenterology Collaborative Hepatocellular Carcinoma Snapshot Audit
STROBE: Strengthening the Reporting of Observational Studies in Epidemiology
T2DM: type 2 diabetes mellitus
TACE: trans‐arterial chemoembolisation

## 1 Introduction

Hepatocellular carcinoma (HCC) accounts for most primary liver cancers and, in the large majority of patients, develops on a background of cirrhosis [1]. Globally, it ranked sixth among cancers by incidence in 2020, with slightly more than 900 000 new diagnoses, and third as a cause of cancer death, with over 800 000 deaths [2]. Because the annual number of deaths approaches the number of new cases, the case fatality of HCC is evidently high.

GLOBOCAN 2022 places liver cancer twentieth among all cancers in Sri Lanka, with an estimated 402 new cases and 308 deaths in that year [3]. A regional analysis of 2020 data ranked Sri Lanka lowest in Asia for both HCC incidence and mortality, with age‐standardised rates (ASR) of 1.2 and 1.1 per 100 000, respectively; across Asia, incidence ranged from 2.1 per 100 000 in Nepal to 85.6 per 100 000 in Mongolia, and mortality from 2.0 to 80.6 per 100 000 in the same two countries [4]. These national figures, however, are modelled rather than measured. Sri Lanka does not have a prospective HCC registry, HCC is not individually notifiable, and the national cancer registry relies on passive notification from pathology laboratories, a route that inevitably misses a tumour that is usually diagnosed on imaging alone. Published information on HCC in the country is limited to reports from a single tertiary centre [5, 6, 7]. Consequently, neither the national incidence nor the clinical profile of HCC in Sri Lanka is known, and it is this gap that the present study set out to fill.

Chronic liver disease is common in Sri Lanka. Community studies have reported non‐alcoholic fatty liver disease (NAFLD) in 33% of an urban population near Colombo and in 18% of a rural plantation‐sector population [8]. Although the liver‐related course of NAFLD is benign in most individuals, approximately one in ten develops progressive fibrosis, with the attendant risks of cirrhosis and HCC [9]. The stage of fibrosis and the burden of metabolic abnormalities are the principal determinants of NAFLD‐related morbidity and mortality [10, 11]. Given this high baseline prevalence and the anticipated rise in its main drivers, obesity and Type 2 diabetes mellitus (T2DM), the contribution of NAFLD to cirrhosis and HCC in Sri Lanka is likely to grow.

No population‐based epidemiological study of HCC has been undertaken in Sri Lanka. The two available hospital‐based studies were both conducted at one centre, the North Colombo Hepatobiliary Centre, and reported on the same cohort in 2013 and 2018 [5, 6]. Meanwhile, practising clinicians have reported anecdotally that HCC is being encountered more often.

To obtain the first national estimate, we therefore undertook a nationwide, cross‐sectional snapshot survey of the incidence and clinical characteristics of newly diagnosed HCC in Sri Lanka.

## 2 Methodology

### 2.1 Study Design and Setting

SLSG CARSA (Sri Lanka Society of Gastroenterology Collaborative Hepatocellular Carcinoma Snapshot Audit) was a nationwide, observational, cross‐sectional snapshot audit of patients newly diagnosed with HCC in Sri Lanka. The study obtained ethical clearance from the Ethics Review Committee of the Faculty of Medicine, University of Kelaniya, Ragama, Sri Lanka (P/58/05/2024). Its design followed the snapshot‐audit approach used in earlier national collaborative studies [12]. The study is reported in accordance with the Strengthening the Reporting of Observational Studies in Epidemiology (STROBE) statement for cross‐sectional studies [13]; the completed STROBE checklist is provided as Supplementary File 1.

The aim was to enumerate and characterise all newly diagnosed cases of HCC in the country over 3 months, with cases contributed from both state and private healthcare institutions in each of the nine provinces.

### 2.2 Sampling Frame, Invitation and Participating Institutions

The sampling frame was constructed from the Ministry of Health register of state‐sector institutions providing gastroenterology, gastrointestinal surgical or oncology services (national, teaching, provincial general and district general hospitals) together with the major private‐sector hospitals offering equivalent services. In total, 40 eligible institutions were identified. All consultant gastroenterologists, gastrointestinal surgeons, hepatobiliary surgeons and oncologists practising in these institutions (90 clinicians) were invited to contribute through the Sri Lanka Society of Gastroenterology, the College of Surgeons of Sri Lanka and the Sri Lanka College of Oncologists and by direct individual approach. Participation was voluntary, and no incentive was offered. An institution was classified as contributing if at least one case was notified from it during the study period; the institutional contribution proportion was calculated as the number of responding institutions divided by the number invited (40), and the clinician contribution proportion as the number of clinicians who notified at least one case divided by the number invited (90). Zero‐case returns were not collected, so these figures are contribution proportions rather than verified response rates. All institutions with a dedicated hepatology or hepatobiliary surgical service and all tertiary oncology centres participated, and at least one contributing institution was located in each of the nine provinces. The flow of invited institutions, contributing institutions, notified cases and included cases is shown in Figure 1.

**FIGURE 1:**
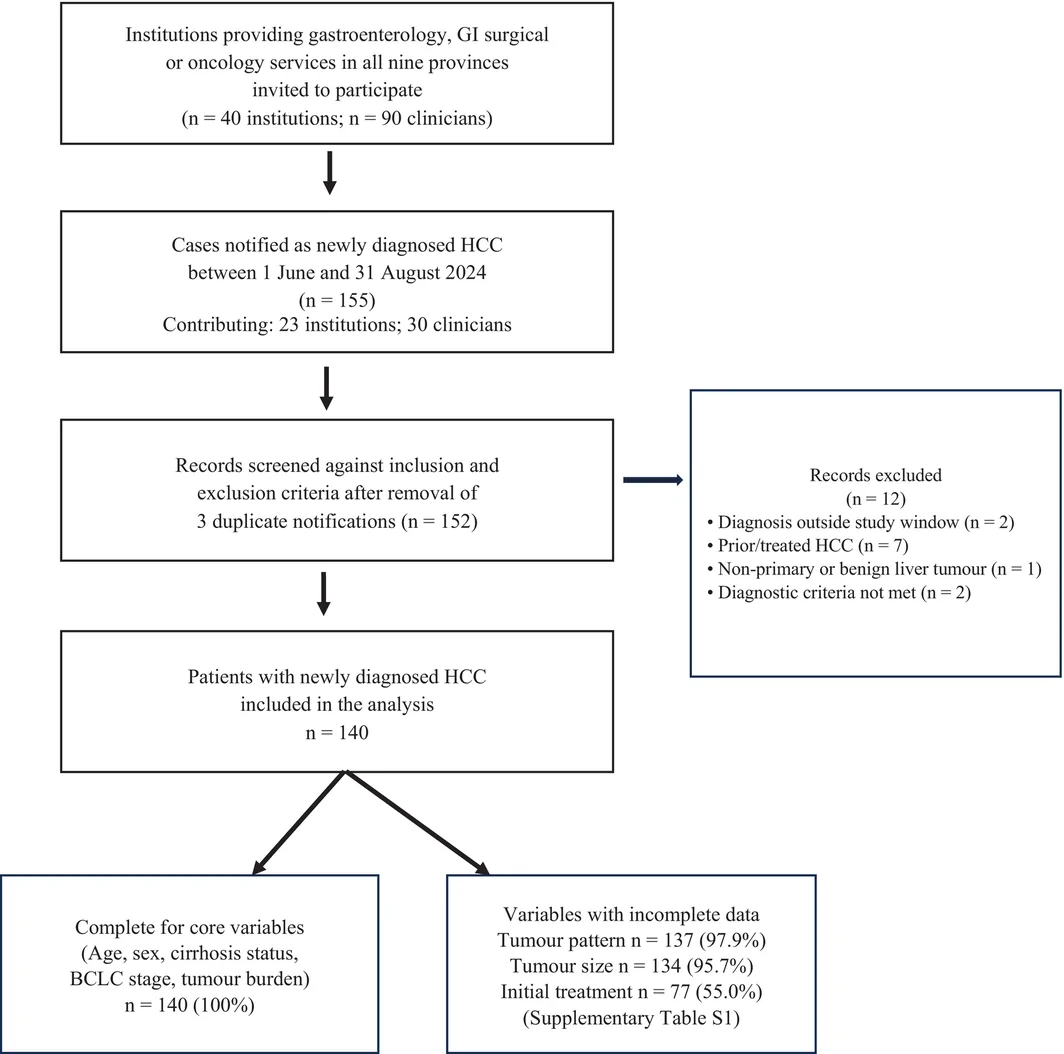
Flow of participating institutions and cases through the SLSG CARSA study.

### 2.3 Eligibility Criteria and Case Definition

Patients were eligible if HCC was newly diagnosed between 01 June 2024 and 31 August 2024; the date on which the diagnosis was confirmed by imaging or histology had to fall within this window. In patients with cirrhosis, the diagnosis was based on dynamic, multiphase, contrast‐enhanced cross‐sectional imaging; histological confirmation was required in patients without cirrhosis and in cirrhotic patients whose imaging was inconclusive [14]. Patients previously diagnosed with, or treated for, HCC were excluded, as were those with secondary (non‐primary) liver cancers or benign liver lesions. A National Identity Card (NIC) number was used as a unique patient identifier to prevent case duplication. Every notification was screened against all records already entered for an identical NIC number and, where the NIC number was not available, against the combination of name, sex, date of birth and date of diagnosis (Figure 1). Because the study was designed as a single cross‐sectional snapshot without follow‐up, a formal sample size calculation was not required.

### 2.4 Variables and Definitions

We collected patient demographics and characteristics, including performance status, underlying liver disease, risk factors, tumour characteristics and initial management approaches. Mode of detection was recorded on the case‐report form as a binary ‘screen detected’ field; the form did not identify surveillance schedules or distinguish formal surveillance from opportunistic screening. Risk factors were recorded by the treating clinician using pre‐specified definitions embedded in the data collection form. Unsafe alcohol use was defined as a self‐reported average consumption exceeding 14 standard units (1 unit = 8 g of ethanol) per week in men or 7 standard units per week in women, sustained for 5 years or more, or a documented clinical diagnosis of alcohol use disorder; formal Alcohol Use Disorders Identification Test (AUDIT) scoring was not undertaken [15]. Smoking was defined as current or former consumption of at least 100 cigarettes in the lifetime. Type 2 diabetes mellitus was defined by a pre‐existing physician diagnosis or the use of glucose‐lowering therapy. Obesity was defined as a body mass index (BMI) of ≥ 27.5 kg/m^2^, in keeping with World Health Organisation recommendations for Asian populations [16], calculated from weight and height measured at the diagnostic admission or clinic visit; waist circumference was not recorded. Cirrhosis was diagnosed on the basis of established histological, radiological (nodular liver contour with features of portal hypertension), endoscopic or elastographic criteria or a combination of these, as documented by the treating clinician.

A patient was classified as having HBV‐ or HCV‐related HCC only when a positive result was documented. Patients in whom testing had not been performed, or in whom the result was not available, were recorded as ‘not tested’ rather than as negative, and the number tested is reported in the Results. Testing for occult hepatitis B infection (antibody to hepatitis B core antigen with HBV DNA) was not part of the protocol.

### 2.5 Data Collection

Cases were recorded on an online REDCap data collection form [17]. Trained research assistants entered each record remotely using clinical information supplied by the treating clinician, who also confirmed on the form that the pre‐specified diagnostic criteria were satisfied; for every case, the relevant radiology or histopathology report was either uploaded to REDCap or transcribed into it verbatim. When essential items were missing, the research assistants telephoned the patient, next of kin or caregiver, using contact details released by the contributing consultant gastroenterologist, gastrointestinal surgeon or oncologist. Information was obtained verbally and, where necessary, from reports shared via the WhatsApp messaging service. Throughout the study period, weekly WhatsApp reminders encouraged potential contributors to notify their newly diagnosed patients. Data collection remained open until 30 September 2024 to accommodate patients who presented late for diagnostic investigations.

### 2.6 Data Quality Assurance

The REDCap instrument incorporated compulsory fields for core variables (age, sex, province, cirrhosis status, basis of diagnosis, tumour burden and BCLC stage), range and logic checks for continuous variables and closed‐ended categorical response options to minimise entry errors. Records were entered by two trained research assistants; a randomly selected sample of records was independently re‐entered by the second assistant and compared field by field to estimate the data entry error rate. Automated completeness reports were generated fortnightly, and any record with a missing core variable generated an electronic query to the reporting clinician. The coordinating team reviewed the uploaded radiology or histopathology report for each case against the pre‐specified diagnostic criteria; when the criteria were not clearly documented, a query was sent to the reporting clinician, and the case was included only after the criteria were confirmed. Discrepancies between the notification form and the source report, biologically implausible values, and unresolved queries were adjudicated independently by two investigators (MAN, VSR), with a third investigator (HJdeS) serving as arbiter when agreement was not reached. Imaging studies and histology slides were not centrally reread, which is acknowledged as a limitation. The database was locked on 30 September 2024 after the final query round.

### 2.7 Handling of Missing Data

No imputation was undertaken. All proportions are presented as complete‐case analyses, and the denominator of non‐missing observations is stated explicitly for every variable in the Results and in Table 2. The number and proportion of missing observations for each variable are reported in Table S1. Variables with more than 20% missing data, including hepatitis B surface antigen (28.6%), anti‐HCV testing (35.7%) and the initial treatment decision (45.0%), are reported descriptively and were not used for any comparative inference.

### 2.8 Data Protection and Ethical Considerations

The collected data were securely stored in a password‐protected cloud‐based repository, with strict access controls to ensure data confidentiality. The approval granted by the Ethics Review Committee (P/58/05/2024) explicitly covered the collection of data from treating clinicians and, where essential items were missing, direct telephone contact with the patient or a nominated next of kin by the study research assistants. Telephone contact was made only after the treating clinician had obtained the patient's or next of kin's agreement to be contacted and had released the contact details. Verbal informed consent was obtained and documented in REDCap at the start of each call. Where a patient or relative chose to share a clinical report electronically, this was done at their own initiative using the end‐to‐end encrypted WhatsApp application on a dedicated, password‐ and biometric‐protected study device; the relevant data were transcribed into REDCap, the images were deleted from the device and from the application within 24 h, application‐level cloud backup was disabled and no identifiable image was retained or transferred to any other system or third party. WhatsApp was used only for clinician reminders and for the receipt of documents volunteered by patients or relatives; it was never used to transmit patient data from the study team. All records held in REDCap were pseudonymised: the NIC number was used solely during data collection to exclude duplicates and was replaced by a study identification number before analysis, with the linking key held separately under restricted access by the study coordinator. No patient‐identifiable information appears in this report.

### 2.9 Statistical Analysis

Both crude and age‐standardised incidence rates (ASRs) were estimated. The crude rate was obtained by dividing the number of incident cases by the 2024 mid‐year population of Sri Lanka (22.067 million) [18]. Age‐specific rates were calculated for the five‐year age bands used in the national mid‐year population estimates (0–4, 5–9, and so on up to 75–79 years, with a final band of ≥ 80 years). The ASR was obtained by direct standardisation to the WHO World Standard Population [19]: each age‐specific rate was multiplied by the weight of the corresponding age band in the standard population, and the weighted rates were summed and divided by the sum of the weights.

$$\mathrm{ASR}=\sum \left(\mathrm{ai}\times \mathrm{wi}\right)\sum \mathrm{wi}\times 100\;000$$

- *ai* = the age‐specific rate for age group *i* in the study population (typically per 100 000).
- *wi* = the weight for age group *i*, based on a standard population (World Standard Population).

Because case ascertainment covered a three‐month window, the incidence was annualised. The observed number of cases in each five‐year age stratum was multiplied by four (12 months ÷ 3 months) to obtain a stratum‐specific annualised count; this was then divided by the corresponding 2024 mid‐year population of that stratum to yield an annualised age‐specific rate, and the age‐specific rates were standardised as described above. The annualised stratum‐specific counts are shown alongside the observed counts in Table 1, so that the derivation is fully transparent.

**TABLE 1:** Distribution of HCC cases according to age groups and the calculation of age‐standardised rates (ASR) of annual incidence.

Age group	Observed cases (3 months)	Annualised cases (×4)	Population (thousands)	Annualised crude rate (per 100 000)	World standard population weight	Weighted contribution to ASR
0–4	0	0	1916	0	0.0886	0.0000
5–9	0	0	1883	0	0.0870	0.0000
10–14	0	0	1886	0	0.0860	0.0000
15–19	1	4	1769	0.23	0.0847	0.0192
20–24	0	0	1773	0	0.0822	0.0000
25–29	0	0	1651	0	0.0793	0.0000
30–34	0	0	1673	0	0.0761	0.0000
35–39	0	0	1516	0	0.0715	0.0000
40–44	1	4	1461	0.27	0.0659	0.0180
45–49	0	0	1383	0	0.0604	0.0000
50–54	3	12	1311	0.92	0.0537	0.0492
55–59	17	68	1143	5.95	0.0455	0.2707
60–64	28	112	986	11.36	0.0372	0.4226
65–69	28	112	678	16.52	0.0296	0.4890
70–74	33	132	442	29.86	0.0221	0.6599
75–79	21	84	303	27.72	0.0152	0.4213
80+	8	32	293	10.92	0.0150	0.1638
**Total**	**140**	**560**	**22 067**	**2.54**	**1.00**	**2.51**

*Note:* Annualised crude incidence rate 2.54 per 100 000 per year (95% CI 2.13–2.99); age‐standardised incidence rate 2.51 per 100 000 per year (95% CI 2.11–2.97). Confidence intervals are derived from the exact Poisson distribution of the observed (unannualised) case count and, for the ASR, by the method of Dobson et al. Population denominators are the 2024 mid‐year estimates of the Department of Census and Statistics, Sri Lanka.

Abbreviations: ASR, age‐standardised rate; HCC, hepatocellular carcinoma.

Annualisation assumes that HCC diagnoses are distributed uniformly across the calendar year. HCC is a chronic disease with no recognised seasonal pattern of onset, and, unlike acute illnesses, the date of its diagnosis is determined by the timing of surveillance imaging or of investigation for symptoms rather than by season. We were, however, unable to test this assumption empirically, because no month‐level national HCC data exist for Sri Lanka. The study quarter (June to August) does not include the two periods in which elective diagnostic activity in Sri Lanka is usually reduced (the April new‐year holidays and late December), so diagnostic activity during the study quarter may have been similar to, or somewhat higher than, that of an average quarter. This possibility could not be tested; if the study quarter was busier than average, annualisation would overestimate the annual incidence. The uniformity assumption is therefore a limitation; its potential effect in either direction is quantified in the seasonality sensitivity analysis described below and considered in the Discussion.

Confidence intervals (CI) for the crude rate were derived from the exact Poisson distribution of the observed (i.e., unannualised) case count, and those for the ASR by the method of Dobson et al. for confidence intervals of weighted sums of Poisson parameters [20]. The statistical uncertainty, therefore, reflects the number of cases actually observed during the three‐month window, not the annualised count. Two pre‐specified sensitivity analyses were performed: (i) a seasonality analysis in which the quarterly case count was varied by ±15% and ±25% to simulate departures from a uniform within‐year distribution; and (ii) an ascertainment analysis in which the incidence was re‐derived under the assumption that the study captured 90%, 80% or 70% of all incident cases nationally. Additional capture scenarios of 60% and 50% were calculated post hoc in response to peer review; no capture fraction was independently validated.

Apart from the incidence estimates, the analysis was purely descriptive. Continuous variables are summarised as means with standard deviations or medians, and categorical variables as counts and percentages with their denominators. The variables summarised were patient characteristics, underlying liver disease, risk factors, tumour characteristics, Barcelona Clinic Liver Cancer (BCLC) stage [21] and the initial management approach, providing a baseline description of how HCC presents and is initially managed in Sri Lanka. No hypothesis testing was undertaken: there was no comparison group, associations between variables (e.g., between aetiology, BMI, mode of detection, stage, treatment and institutional resources) were not examined statistically, and comparisons with other published series are descriptive only. Data were analysed using JASP 0.18.3.0; incidence rates, confidence intervals, and sensitivity analyses were computed in R version 4.3.1.

## 3 Results

### 3.1 Participating Institutions and Flow of Cases

Of the 40 eligible institutions invited, 23 (57.5%) contributed at least one case, and 30 of the 90 invited clinicians (33.3%) did so as well (Figure 1). Contributing institutions were located in all nine provinces. Without nil‐case returns, an institution notifying no cases could not be distinguished from one that did not participate. A total of 155 notifications were received during the study period. Of these, 15 were excluded (3 duplicate notifications identified by NIC number, 2 with a date of diagnosis outside the study window, 7 with previously diagnosed or previously treated HCC, 1 with non‐primary or benign liver lesions and 2 in whom the pre‐specified diagnostic criteria were not met), leaving 140 patients for analysis (Figure 1). Core demographic and cirrhosis‐status data were complete for all 140 patients. BCLC‐stage counts were reported for the whole cohort in the original study tables but could not be reconstructed from the retained analytical export.

### 3.2 Incidence

Over the three‐month study period, 140 patients from the nine provinces of Sri Lanka were reported to have newly diagnosed HCC. Annualised, this corresponds to a crude incidence rate of 2.54 per 100 000 population per year (95% CI 2.13–2.99) and an ASR of 2.51 per 100 000 population per year (95% CI 2.11–2.97) (Table 1). In the seasonality sensitivity analysis, varying the quarterly case count by ±15% gave an ASR range of 2.14–2.89 per 100 000 and by ±25% a range of 1.89–3.14 per 100 000. In the ascertainment sensitivity analysis, if the study captured 90%, 80% or 70% of all incident cases nationally, the corresponding ASRs would be 2.79, 3.14 and 3.59 per 100 000, respectively. Exploratory scenarios assuming 60% and 50% case capture give corresponding ASRs of 4.18 and 5.02 per 100 000; these are hypothetical, not observed, capture fractions.

### 3.3 Presentation

A total of 65 (46.4%) of HCC patients were recorded as screen‐detected, while 75 (53.6%) were recorded as not screen‐detected. Among 120 patients with cirrhosis, 55 (45.8%) were recorded as screen‐detected; this also applied to 10 of 20 (50.0%) without cirrhosis. The presenting symptoms included weight loss in 57 (40.7%), anorexia in 52 (37.1%), abdominal pain in 49 (35.0%), abdominal distension in 45 (32.1%), fever in 21 (15.0%) and nausea in 20 (14.3%) patients, respectively (denominator 140 for all; patients could report more than one symptom).

### 3.4 Patient Characteristics

Of the 140 patients, 110 were male (78.6%) and 30 were female (21.4%), yielding a 3.6:1 male‐to‐female ratio. The mean age at diagnosis of HCC was 67.4 years (SD = 8.9 years); the age distribution is shown in Table 1. In total, 120 (85.7%) patients with HCC had underlying cirrhosis, while 20 (14.3%) had no underlying cirrhosis. Among patients with HCC and cirrhosis (*n* = 120), the shares in Child‐Turcotte‐Pugh (CTP) Classes A, B, and C were 75 (62.5%), 41 (34.2%) and 4 (3.3%), respectively.

### 3.5 Tumour Characteristics

HCCs were classified as single nodular in 60 patients (43.8%), followed by paucinodular (2 or 3 nodules) in 39 patients (28.5%), multinodular (more than three nodules) in 25 patients (18.2%) and diffuse type in 13 patients (9.5%); the tumour pattern was recorded for 137 of 140 patients (97.9%), and these percentages use 137 as the denominator. At diagnosis, HCCs in 61 (45.5%) patients were reported to have a tumour size of 2–5 cm. This was followed by tumour sizes 5–10 cm in 34 (25.4%), < 2 cm in 17 (12.7%) and more rarely > 10 cm in 22 (16.4%) patients, respectively; the size of the largest lesion was recorded for 134 of 140 patients (95.7%) and these percentages use 134 as the denominator. The median tumour size was 4.5 cm (*n* = 134).

In 117 (83.6%) patients, there was no tumour invasion. In 23 (16.4%) patients, HCCs showed tumour invasion [21 (15.0%) to the portal vein, 6 (4.3%) to the hepatic vein, 8 (5.7%) to the inferior vena cava and 1 (0.7%) to the bile ducts; individual sites sum to more than 23 because several patients had invasion at more than one site]. In 122 (87.1%) patients, there was no extra‐hepatic spread. In 18 (12.9%) patients, HCCs showed extrahepatic spread [5 (3.6%) to the lungs, 5 (3.6%) to the peritoneum and 11 (7.9%) elsewhere; again, several patients had more than one site of spread]. The proportions of HCC patients in BCLC Stages 0, A, B, C and D were 17 (12.1%), 39 (27.9%), 43 (30.7%), 23 (16.4%) and 18 (12.9%), respectively (*n* = 140). Taken together, 56 patients (40.0%) presented at BCLC Stage 0 or A, 66 (47.1%) at Stage B or C and 18 (12.9%) at stage D.

### 3.6 Risk Factors Recorded in Patients With HCC

Cigarette smoking was recorded in 63 (45.0%) and unsafe alcohol use in 88 (62.9%) of the 140 patients, and Type 2 diabetes mellitus in 93 (66.4%). BMI was recorded for 130 of the 140 patients (92.9%) and 18 of these 130 patients (13.8%) were obese. No positive HBsAg or anti‐HCV results were documented among those tested. Hepatitis B surface antigen was documented in 100 patients (71.4%), and antibody to hepatitis C virus in 90 patients (64.3%); all documented results were negative. Testing status was unavailable for the remaining patients, who were classified as “not tested” rather than “negative”.

### 3.7 Initial Treatment Offered

Initial treatment decisions were available for 77 (55.0%) patients; the treatment field was not completed for the remaining 63 patients (45.0%) and the percentages below use 77 as the denominator. Systemic treatment with sorafenib was the most commonly offered modality to 30 patients (39.0%). Transarterial chemoembolisation (TACE) was offered to 20 (26.0%), percutaneous ethanol injection (PEI) to 11 (14.3%), surgical resection to 9 (11.7%) and microwave ablation to 7 (9.1%) patients as the initial treatment modality. No patient was offered liver transplantation as an initial treatment.

Key findings of the study are summarised in Table 2, and the completeness of each variable in Table S1.

**TABLE 2:** Summary of key findings in the study.

Category	Parameter	Number (%)	Denominator (*n* with data)
Detection	Screen‐detected (recorded)	65 (46.4%)	140
	Not screen‐detected (recorded)	75 (53.6%)	140
Presenting Symptoms	Weight loss	57 (40.7%)	140
	Anorexia	52 (37.1%)	140
	Abdominal pain	49 (35.0%)	140
	Abdominal distension	45 (32.1%)	140
	Fever	21 (15.0%)	140
	Nausea	20 (14.3%)	140
Demographics	Males	110 (78.6%)	140
	Females	30 (21.4%)	140
	Mean age	67.4 ± 8.9 years	140
Cirrhosis Status	With cirrhosis	120 (85.7%)	140
	Without cirrhosis	20 (14.3%)	140
CTP Class	A	75 (62.5%)	120
	B	41 (34.2%)	120
	C	4 (3.3%)	120
Tumour Pattern	Single nodular	60 (43.8%)	137
	Paucinodular (2, 3)	39 (28.5%)	137
	Multinodular (> 3)	25 (18.2%)	137
	Diffuse	13 (9.5%)	137
Tumour Size	< 2 cm	17 (12.7%)	134
	2–5 cm	61 (45.5%)	134
	5–10 cm	34 (25.4%)	134
	> 10 cm	22 (16.4%)	134
	Median size	4.5 cm	134
BCLC Stage	0	17 (12.1%)	140
	A	39 (27.9%)	140
	B	43 (30.7%)	140
	C	23 (16.4%)	140
	D	18 (12.9%)	140
Invasion	No invasion	117 (83.6%)	140
	Portal vein	21 (15.0%)	140
	Hepatic vein	6 (4.3%)	140
	Inferior vena cava	8 (5.7%)	140
	Bile ducts	1 (0.7%)	140
Extra‐hepatic Spread	Lungs	5 (3.6%)	140
	Peritoneum	5 (3.6%)	140
	Other sites	11 (7.9%)	140
Associated Conditions	Type 2 diabetes	93 (66.4%)	140
	Unsafe alcohol use	88 (62.9%)	140
	Cigarette smoking	63 (45.0%)	140
	Obesity	18 (13.8%)	130
	Hepatitis B, C	0 (0%)	100, 90
Initial Treatment	Sorafenib	30 (39.0%)	77
	TACE	20 (26.0%)	77
	PEI	11 (14.3%)	77
	Surgical resection	9 (11.7%)	77
	Microwave ablation	7 (9.1%)	77
	Liver transplantation	0 (0%)	77

*Note:* A denominator column has been added so that every percentage can be verified. Percentages for tumour pattern, tumour size, CTP class and initial treatment use the number of patients with that variable recorded, not the full cohort of 140. Patients could have more than one presenting symptom, more than one site of vascular invasion and more than one site of extra‐hepatic spread, so these categories are not mutually exclusive and do not sum to the denominator. The percentage for BCLC stage D has been corrected from 5.7% to 12.9% (18/140); the previously published value was a typographical error. Counts have been added to the initial treatment rows. Completeness of every variable is tabulated in Table S1.

Abbreviations: BCLC, Barcelona Clinic Liver Cancer; CTP, Child‐Turcotte‐Pugh; PEI, percutaneous ethanol injection; TACE, transarterial chemoembolisation.

## 4 Discussion

This nationwide, cross‐sectional snapshot survey yielded an estimated ASR for HCC of 2.51 per 100 000 population per year (95% CI 2.11–2.97), more than double the figure reported for 2020 [4]. Just over half of the tumours (53.6%) were not recorded as screen‐detected. Patients were predominantly male (78.6%), with a mean age of 67.4 years, and 85.7% had underlying cirrhosis, most often CTP class A (62.5%). The commonest presentation was a single nodule (43.8%) measuring 2–5 cm (45.5%). Tumour invasion was present in 16.4% and extra‐hepatic spread in 12.9% at diagnosis, and BCLC Stages A (27.9%) and B (30.7%) predominated. Diabetes (66.4%) and unsafe alcohol use (62.9%) were the most frequently recorded risk factors. Among patients with a recorded treatment decision, sorafenib (39.0%) and TACE (26.0%) were the most frequent initial treatments offered.

This is the first nationwide study to quantify and describe HCC in Sri Lanka. In the most recent regional analysis of GLOBOCAN 2020 data, Sri Lanka had the lowest estimated HCC incidence in Asia, at 1.20 per 100 000 population [4], a figure that may partly reflect under‐detection and under‐reporting in the absence of robust epidemiological surveillance. The ASR of 2.51 per 100 000 obtained in the present study is twice that estimate and, although probably still an underestimate, points to a considerable and previously under‐recognised burden of HCC in Sri Lanka.

These estimates should be read as the best currently available approximation of the national burden, rather than as a definitive incidence figure, for three reasons. First, a three‐month snapshot samples a single quarter and is annualised on the assumption that diagnoses are distributed uniformly across the year; even under that assumption the estimate carries appreciable statistical uncertainty (95% CI 2.11–2.97), and any genuine seasonal variation would widen it further, as the sensitivity analysis shows (ASR range 1.89–3.14 for a ±25% variation in quarterly case load). The direction of any seasonal bias is unknown. If the study quarter was busier than an average quarter, which is possible because it excluded the main holiday periods but could not be verified, the annualised estimate would be biassed upwards; even a 25% overestimate, however, would leave the ASR (1.89 per 100 000) above the modelled 2020 figure. Second, and more importantly, the comparison with the 2020 estimate of 1.20 per 100 000 is not a like‐for‐like comparison of two surveillance measurements: the earlier figure was modelled in the absence of national HCC surveillance, whereas the present figure derives from active, clinician‐led case notification. A substantial part of the apparent doubling is therefore likely to reflect better case ascertainment rather than a true rise in incidence, and the present data cannot distinguish between these explanations. Third, the present estimate is itself almost certainly conservative: the ascertainment sensitivity analysis suggests that even 70% national capture would place the true ASR at about 3.59 per 100 000. The safest interpretation is that the burden of HCC in Sri Lanka is substantially higher than previously assumed, probably around twice as high, and that a temporal trend cannot be established from a single snapshot.

Table 3 sets the principal findings of the present study alongside those of the earlier single‐centre Sri Lankan cohort [6] and of published regional data, including INSIGHT, a longitudinal multicentre cohort that described real‐world HCC diagnosis and management across the Asia‐Pacific region [22]. These comparisons are descriptive only. INSIGHT pooled patients from several Asia‐Pacific countries with markedly different HCC aetiology (predominantly hepatitis B), surveillance coverage, treatment availability and health‐financing arrangements and used a longitudinal cohort design with different eligibility criteria and case‐mix from the present cross‐sectional snapshot. No formal statistical comparison between the datasets was undertaken, and none would be valid; Table 3 is therefore presented as a juxtaposition of published figures rather than as a hypothesis test. Accordingly, the differences discussed below are numerical differences between published figures and should not be read as statistically tested differences.

**TABLE 3:** Quantitative comparison of the present study with the previous Sri Lankan cohort and published regional data.

Characteristic	SLSG CARSA, Sri Lanka, 2024 (*n* = 140)	Previous Sri Lankan cohort (single centre)	Regional comparators
Design and period	Nationwide cross‐sectional snapshot, Jun–Aug 2024	Single‐centre prospective cohort, tertiary referral centre [6]	INSIGHT: multinational prospective cohort, Asia‐Pacific [22]
ASR of incidence (per 100 000/year)	2.51 (95% CI 2.11–2.97)	Not applicable (hospital series)	Asia 2020: 2.1 (Nepal) to 85.6 (Mongolia); Sri Lanka 1.20 [4]
Mean age at diagnosis (years)	67.4 (SD 8.9)	64 [6]	China and Taiwan 63 [4]
Male (%)	78.6	86 [6]	83.5 (INSIGHT) [22]
Underlying cirrhosis (%)	85.7	78 [6]	80–90 (global) [1]
HBV‐ or HCV‐related HCC (%)	0 (HBsAg 0/100; anti‐HCV 0/90)	Not reported	Up to 75 of all HCC globally [1]
Single nodular tumour (%)	43.8 (60/137)	42.4 [6]	Not reported
Diffuse‐type tumour (%)	9.5 (13/137)	16.7 [6]	Not reported
Median size of the largest tumour (cm)	4.5 (*n* = 134)	6.9 [6]	Not reported
Macrovascular/local invasion (%)	16.4	28.5 [6]	Not reported
Extra‐hepatic spread (%)	12.9	6.9 [6]	Not reported
BCLC stage 0 or A (%)	40.0	Not reported	Japan and Taiwan > 70 [23]
BCLC stage B or C (%)	47.1	Not reported	Korea and China > 60 [23]
Surgical resection as initial treatment (%)	11.7 (9/77)	14 [6]	34 (INSIGHT) [22]
TACE as initial treatment (%)	26.0 (20/77)	35 [6]	Not reported
Ablation (PEI or microwave) as initial treatment (%)	23.4 (18/77)	PEI 8; radiofrequency ablation 6 [6]	Not reported
Systemic therapy (sorafenib) as initial treatment (%)	39.0 (30/77)	Not reported	Not reported
Liver transplantation as initial treatment (%)	0	Not reported	< 1 (INSIGHT); 1 (China and Taiwan) [22]

Abbreviations: ASR, age‐standardised rate; BCLC, Barcelona Clinic Liver Cancer; CI, confidence interval; HBV, hepatitis B virus; HCC, hepatocellular carcinoma; HCV, hepatitis C virus; PEI, percutaneous ethanol injection; TACE, transarterial chemoembolisation.

The mean age at diagnosis in the present study (67.4 years) was a few years higher than that reported in the earlier Sri Lankan cohort (64 years) and in China and Taiwan (63 years) [4, 6]. The proportion of men (78.6%) was lower than in the earlier Sri Lankan cohort (86%) and in INSIGHT (83.5%), whereas the proportion with underlying cirrhosis (85.7%) was higher than in the earlier cohort (78%) and within the 80%–90% range reported globally [1].

Diabetes (66.4%) and unsafe alcohol use (62.9%) were the most frequently recorded risk factors in this cohort. Because the study had no comparison group, these figures describe how common each factor was among patients with HCC; they do not establish an association with HCC or quantify the risk attributable to it. The recorded prevalence of obesity (18 of the 130 patients with BMI recorded, 13.8%) is, however, difficult to reconcile with a population in which a third of urban adults have fatty liver disease [8] and two‐thirds of the present cohort had Type 2 diabetes. Measurement is a more likely explanation than biology, although this could not be examined formally: BMI was recorded at a single diagnostic encounter, was unavailable for 10 patients (7.1%), and may have been lowered at presentation by tumour‐ and cirrhosis‐related weight loss, sarcopenia and the use of ascites‐adjusted weight. Waist circumference, which is a better marker of metabolic risk in South Asians, was not recorded. The prevalence of obesity reported here should therefore be regarded as a floor rather than an estimate of metabolic risk in this population, and future work should prospectively record measured height, weight, and waist circumference and apply Asian‐specific thresholds. No positive HBsAg or anti‐HCV results were documented among those tested. This contrasts sharply with the global picture, in which chronic hepatitis B and C account for up to 75% of HCC [1], but is consistent with Sri Lanka's low prevalence of chronic hepatitis B and C [24, 25].

The absence of documented virus‐related HCC nevertheless deserves careful scrutiny, because a count of exactly zero is unusual even in low‐prevalence settings. Two explanations must be weighed. The finding is biologically plausible: the seroprevalence of chronic hepatitis B and C in Sri Lanka is among the lowest in Asia [24, 25], hepatitis B vaccination has been part of the national expanded programme on immunisation since 2003; injecting drug use is uncommon; and the two previous hospital‐based Sri Lankan series also found viral aetiology to be rare [5, 6]. Against this, the ascertainment artefact cannot be excluded. Serological testing was not universal, and patients who were not tested were classified as “not tested” rather than negative; occult hepatitis B infection was not sought and would not have been detected by hepatitis B surface antigen testing alone; and reporting depended on the treating clinician transcribing a result rather than on central serology. Even if all 140 patients had been tested and all were negative, the upper limit of the exact two‐sided 95% confidence interval for an observed zero would still be 2.6%; for the 100 patients actually tested for hepatitis B surface antigen and the 90 tested for antibody to hepatitis C virus, the corresponding upper limits are 3.6% and 4.0%, respectively. We therefore interpret the zero documented viral cases cautiously, as incomplete testing prevents exclusion of viral aetiology. This has direct policy relevance: a national HCC control strategy for Sri Lanka cannot be modelled on the viral‐hepatitis‐driven programmes that dominate elsewhere in Asia.

Single‐nodular tumours accounted for 43.8% and paucinodular tumours for 28.5% of cases in the present study; the former proportion is close to the 42.4% reported in the earlier Sri Lankan cohort [6]. Multinodular and diffuse‐type tumours made up 18.2% and 9.5%, respectively, the latter being lower than the 16.7% reported previously [6]. Most tumours measured 2–5 cm at diagnosis (45.5%), and the median diameter was 4.5 cm, compared with 6.9 cm in the earlier cohort [6].

Compared with the earlier cohort [6], local invasion was less frequent (16.4% vs. 28.5%) and extra‐hepatic spread more frequent (12.9% vs. 6.9%) at presentation. BCLC Stages A (27.9%) and B (30.7%) accounted for most patients. Published regional data show that more than 70% of HCC in Japan and Taiwan is diagnosed at BCLC Stage 0 or A, stages with a favourable prognosis, whereas in Korea and China more than 60% of patients present at Stage B or C, with correspondingly worse outcomes [23]. The Sri Lankan distribution, therefore, sits between these two patterns: 40.0% of patients were detected at BCLC stage 0 or A, which is well below the Japanese and Taiwanese figures, while 47.1% presented at Stage B or C and a further 12.9% at Stage D, when curative options are no longer available.

### 4.1 Why Were Fewer Than Half of the Cases Recorded as Screen‐Detected?

Only 46.4% of patients were recorded as screen‐detected, despite cirrhosis being present in 85.7%. This flag does not establish participation in guideline‐based HCC surveillance. The present study was not designed to identify the reasons for this, and none of the explanations that follow was tested in our data; they are hypotheses, drawn from published literature and local clinical experience, that require formal evaluation. First, cirrhosis in Sri Lanka is often recognised for the first time only at decompensation, so many patients may not have been enrolled in surveillance before HCC developed. Second, there is no national HCC surveillance programme, no funded recall mechanism and no ultrasound capacity ring‐fenced for surveillance, so enrolment depends on individual clinicians' practise and on patients' ability to attend and pay for six‐monthly imaging. Third, patients with alcohol‐related and metabolic liver disease may engage less consistently with long‐term follow‐up than those with viral hepatitis, who are retained within antiviral treatment programmes; if so, the rarity of viral aetiology in Sri Lanka would remove a mechanism that sustains surveillance elsewhere in Asia. We did not, however, compare surveillance participation across aetiologies. Fourth, ultrasound is recognised to have limited sensitivity for early HCC, particularly in patients with obesity or a coarse, nodular liver [14]; because obesity was uncommon in this cohort and the performance of surveillance could not be assessed in a cross‐sectional design, the contribution of this factor here is uncertain. Finally, 14.3% of patients had no cirrhosis and would not have met the criteria for surveillance under any current guideline.

An initial treatment decision was available for only 77 (55.0%) patients, which limits how far these findings can be generalised. Among these, sorafenib was offered to 39.0% and TACE to 26.0%. Potentially curative options were offered less often: PEI to 14.3%, surgical resection to 11.7% and microwave ablation to 9.1%. In the earlier Sri Lankan cohort, TACE was the commonest treatment (35%), followed by curative surgery (14%), PEI (8%) and radiofrequency ablation (6%) [6]. The proportion offered resection in the present study was lower than the 34% resection rate reported in INSIGHT [22]; differences in stage at presentation, access to surgery and treatment practise may contribute, but none of these explanations could be examined in the present data. No patient in the present study was offered liver transplantation, compared with 1% in China and Taiwan and less than 1% in INSIGHT [22]. The treatment field was missing for 45.0% of patients. If, as seems plausible but was not assessed, missingness was more frequent in institutions without on‐site interventional or surgical services, the recorded proportions would over‐represent the practise of better‐resourced centres; they should therefore not be taken as a reliable description of national treatment patterns.

### 4.2 Limitations

This study has several limitations, which we group as follows.

#### 4.2.1 Under‐Ascertainment of Cases

The ASR reported here is most likely underestimated, given the possibility of missing early asymptomatic HCCs in patients not under HCC surveillance, under‐reporting, patients treated by non‐specialists, and patients seeking alternative medical therapies. We were also unable to capture patients by interrogating all contrast‐enhanced, cross‐sectional abdominal imaging performed nationally, and no independent data source (national cancer registry, pathology database or death certification) was available against which the completeness of case capture could be validated. The ascertainment sensitivity analysis illustrates possible effects under hypothetical capture fractions, without measuring actual ascertainment:

#### 4.2.2 Voluntary Participation and Reporting Bias

Participation was voluntary, and clinicians with a specialist interest in liver disease, or those working in better‐resourced institutions, may have been more likely both to participate and to report completely. This is likely to bias the cohort towards patients managed in specialist units, and therefore potentially towards earlier stage at diagnosis and greater treatment intensity than in the country as a whole.

#### 4.2.3 Short Study Duration

A three‐month window necessarily yields a small number of events (140), which both limits precision (95% CI 2.11–2.97 for the ASR) and prevents any assessment of within‐year variation. Annualisation rests on an assumption of uniformity across the year that we could not test empirically. Because the study quarter excluded the holiday periods in which diagnostic activity is usually reduced, it may have been busier than an average quarter; if so, annualisation will have biassed the incidence estimate upwards, partly offsetting the under‐ascertainment described above. The seasonality sensitivity analysis indicates the likely size of this effect.

#### 4.2.4 Descriptive Design

Apart from the incidence estimates, the analysis was descriptive, with no comparison group and no hypothesis testing. The proportions reported for risk factors, mode of detection, stage and treatment therefore describe this cohort but cannot establish associations or explain the patterns observed, and the explanations offered in the Discussion should be regarded as hypotheses for future analytical studies.

#### 4.2.5 No Central Validation of Diagnoses

Imaging studies and histology slides were not centrally re‐read. Although the reporting clinician confirmed that pre‐specified diagnostic criteria were met and the source report was reviewed by the coordinating team, misclassification at the level of the reporting institution cannot be excluded.

#### 4.2.6 Missing Data

The initial treatment decision was recorded for only 55.0% of patients, and tumour pattern and size for 97.9% and 95.7%, respectively (Table S1). Missingness in the treatment field may well be systematic rather than random, and treatment patterns must therefore be interpreted with caution. Patient‐level BCLC stage assignments and ECOG performance‐status values were not available in the retained analytical export, preventing independent verification of the reported stage C/D distribution. Potential staging misclassification therefore cannot be excluded.

#### 4.2.7 No Survival or Follow‐Up Data

By design, this was a cross‐sectional snapshot with no longitudinal component. We are therefore unable to report treatment received (as opposed to treatment decided), stage‐specific survival, or the effect of surveillance detection on outcome: the outcomes that matter most for policy.

#### 4.2.8 Incomplete Risk Factor Characterisation

Alcohol intake was self‐reported and not quantified using a validated instrument such as AUDIT; BMI was recorded at a single encounter and was unavailable for 7.1% of patients; waist circumference and formal metabolic phenotyping were not undertaken; and viral serology was not universally performed. No mutually exclusive attribution of HCC to alcohol‐related, metabolic, viral or cryptogenic aetiology was available.

#### 4.2.9 Small Numbers Within Strata

The number of patients (*n* = 140) was lower than in previous studies conducted in Sri Lanka, and estimates within individual age strata and subgroups are correspondingly imprecise.

Despite these limitations, the present estimate is the best available for Sri Lanka and provides a defensible baseline. Long‐term prospective collection of national data through a national HCC registry, with survival follow‐up and linkage to the national cancer registry, is needed to characterise the disease, its treatment and its outcomes more reliably.

### 4.3 Implications for National Screening and Surveillance Policy

These observations have direct implications for policy. Because viral testing was incomplete and aetiology was not adjudicated, surveillance policy should be informed by locally verified risk profiles rather than assuming that risk factors represent mutually exclusive causes. A pragmatic national strategy would need to: (i) define and enumerate the at‐risk population, namely all patients with cirrhosis of any cause and, potentially, selected non‐cirrhotic patients with metabolic dysfunction‐associated steatotic liver disease and advanced fibrosis; (ii) embed six‐monthly ultrasound, with or without alpha‐fetoprotein, into existing clinic workflows with an active recall mechanism rather than relying on opportunistic re‐attendance; (iii) address the substantial proportion of HCC arising outside cirrhosis (14.3% in this cohort), for whom no surveillance criteria currently apply; (iv) expand access to curative modalities, given that only 11.7% of patients with a recorded treatment decision were offered resection and none were offered transplantation and (v) link case detection to a prospective national HCC registry, so that the stage shift and survival gain achieved by any such programme can be measured. The present study provides the baseline against which such a programme could be evaluated.

## 5 Conclusion

This nationwide, cross‐sectional snapshot survey provides the first national estimate of HCC incidence in Sri Lanka: an ASR of 2.51 per 100 000 per year (95% CI 2.11–2.97), approximately twice the previously reported modelled estimate. As the study population was confined to Sri Lanka, these findings should not be extrapolated to the wider South Asian region.

In SLSG CARSA, most cases were not recorded as screen‐detected. Patients were mainly older men, and cirrhosis was highly prevalent. Diabetes and unsafe alcohol use were the most frequently recorded risk factors, and no positive HBV or HCV serology was documented among those tested. Liver function was usually well preserved (Child‐Turcotte‐Pugh Class A), and tumours were most often single nodules of moderate size, with invasion or extra‐hepatic spread in a minority. Although BCLC Stages A and B predominated, fewer than half were recorded as screen‐detected, and only a minority were offered potentially curative treatment.

These findings argue for three national priorities. First, national surveillance and reporting for HCC must be strengthened, including systematic notification of radiologically diagnosed HCC to the national cancer registry, so that incidence is measured rather than modelled. Second, earlier detection requires a structured surveillance programme for patients with cirrhosis and other high‐risk groups, with active recall, informed by locally verified aetiological data rather than untested assumptions about the causes of HCC. Third, and most importantly, a prospective national HCC registry with survival follow‐up should be established to quantify the true burden and outcomes of the disease and to guide healthcare planning and resource allocation in Sri Lanka.

## Author Contributions

**Madunil Anuk Niriella:** conceptualization, investigation, writing – original draft, methodology, validation, writing – review and editing, formal analysis, project administration, supervision, funding acquisition, resources, data curation. **Viranga Sathsarani Ranathunga:** data curation, formal analysis, writing – original draft, investigation. **Sanduni Nirasha:** investigation, data curation, formal analysis, writing – original draft. **Rohan Chaminda Siriwardana:** writing – review and editing, data curation, investigation. **Kuleesha Senarath Kodisinghe:** data curation, writing – review and editing, investigation. **Sujeewa Ravindu Kumarasena:** data curation, writing – review and editing, investigation. **Tharanga Liyanaarchchi:** investigation, writing – review and editing, data curation. **Suchintha Bandara Tillakaratne:** investigation, data curation, writing – review and editing. **Hasitha Wijewantha:** investigation, data curation, writing – review and editing. **Uditha Bandara Dassanayake:** writing – review and editing, data curation, investigation. **Vithiya Rishikesawan:** investigation, writing – review and editing, data curation. **Chamila Kumara Ranawaka:** investigation, writing – review and editing, data curation. **Anuradha Kajendran:** investigation, writing – review and editing, data curation. **Karthiha Balendran:** investigation, writing – review and editing, data curation. **Sanjeewa Aryasingha:** investigation, writing – review and editing, data curation. **Duminda Subasinghe:** data curation, writing – review and editing, investigation. **Nilesh Fernandopulle:** investigation, writing – review and editing, data curation. **Sudual Mananjala Senanayake:** data curation, writing – review and editing, investigation. **Anuradha Supun Dassanayake:** investigation, writing – review and editing, data curation. **Ishan de Zoysa:** data curation, writing – review and editing, investigation. **Chinthaka Viduranga de Silva:** data curation, writing – review and editing, investigation. **Chamila Subasinghe:** writing – review and editing, investigation, data curation. **Sumedha Udumalagala:** investigation, writing – review and editing, data curation. **Supun Kulathunga:** investigation, writing – review and editing, data curation. **Tilan Aponso:** conceptualization, writing – review and editing, data curation. **Vadivel Vijitharan:** writing – review and editing, data curation, investigation. **Isuru Upanishad:** investigation, writing – review and editing, data curation. **Nandana Dinamithra:** investigation, data curation, writing – review and editing. **Arunasalam Pathmeswaran:** investigation, writing – review and editing, data curation. **Hithanadura Janaka de Silva:** investigation, writing – review and editing, supervision, data curation, methodology.

## Funding

This study received a grant from the Sri Lanka Society of Gastroenterology, Colombo, Sri Lanka.

## Disclosure

The study is reported in accordance with the STROBE statement for cross‐sectional studies. The completed STROBE checklist is submitted as Supplementary File 1.

## Ethics Statement

The study obtained ethical clearance from the Ethics Review Committee of the Faculty of Medicine, University of Kelaniya, Ragama, Sri Lanka (P/58/05/2024). The approval covered the collection of clinical data from treating clinicians and, where essential items were missing, direct telephone contact with the patient or a nominated next of kin. The study was conducted in accordance with the Declaration of Helsinki.

## Consent

Informed consent was obtained from the participants. Verbal informed consent was obtained and documented in REDCap from every patient or nominated next of kin who was contacted directly by the study team, in accordance with the approval granted by the Ethics Review Committee.

## Conflicts of Interest

The authors declare no conflicts of interest.

## Supporting information


**Table S1:** Completeness of data for each study variable (*N* = 140).

**Data S1:** STROBE statement—checklist of items that should be included in reports of cross‐sectional studies.

## Data Availability

The data supporting this study's findings are available on request from the corresponding author, subject to the approval of the Ethics Review Committee of the Faculty of Medicine, University of Kelaniya, Ragama, Sri Lanka. The data are not publicly available due to privacy or ethical restrictions.
