# Supplementary material for: Sri Lanka Society of Gastroenterology Collaborative Hepatocellular Carcinoma Snapshot Audit (SLSG CARSA): A Nationwide, Cross‐Sectional Snapshot Study
Source: Cancer Rep (Hoboken). 2026 Nov 5;9(11):e70746. doi: 10.1002/cnr2.70746 (PMC13739420; doi:10.1002/cnr2.70746)
Supplement: Supplementary file 2 [file CNR2-9-e70746-s002.docx]

**Supplementary material**

## Supplementary Table S1 – Completeness of data for each study variable (N = 140)

| **Variable** | **Records with data, n (%)** | **Missing, n (%)** | **Handling in analysis** |
| --- | --- | --- | --- |
| Age | 140 (100) | 0 (0) | Complete case |
| Sex | 140 (100) | 0 (0) | Complete case |
| Province of diagnosis | 140 (100) | 0 (0) | Complete case |
| Mode of detection (screen-detected vs other) | 140 (100) | 0 (0) | Complete case |
| Presenting symptoms | 140 (100) | 0 (0) | Complete case |
| Cirrhosis status | 140 (100) | 0 (0) | Complete case |
| Child-Turcotte-Pugh class (of 120 with cirrhosis) | 120 (100) | 0 (0) | Complete case |
| Basis of diagnosis (imaging/histology) | 140 (100) | 0 (0) | Complete case |
| BCLC stage | 140 (100) | 0 (0) | Complete case |
| Vascular invasion | 140 (100) | 0 (0) | Complete case |
| Extra-hepatic spread | 140 (100) | 0 (0) | Complete case |
| Tumour pattern (nodularity) | 137 (97.9) | 3 (2.1) | Complete case; denominator 137 |
| Size of the largest tumour | 134 (95.7) | 6 (4.3) | Complete case; denominator 134 |
| Type 2 diabetes mellitus | 140 (100) | 0 (0) | Complete case |
| Unsafe alcohol use | 140 (100) | 0 (0) | Complete case |
| Cigarette smoking | 140 (100) | 0 (0) | Complete case |
| Body mass index/obesity | 130 (92.9) | 10 (7.1) | Complete case; denominator 130 |
| Hepatitis B surface antigen | 100 (71.4) | 40 (28.6) | Untested recorded as “not tested” |
| Antibody to the hepatitis C virus | 90 (64.3) | 50 (35.7) | Untested recorded as “not tested” |
| Initial treatment decision | 77 (55.0) | 63 (45.0) | Descriptive only; not used for inference |

BCLC – Barcelona Clinic Liver Cancer. No imputation was performed; all analyses are complete-case analyses with the denominator stated.
